# Ethnobotanical survey of the traditional antiparasitic use of medicinal plants in humans and animals in Laghouat (Southern Algeria)

**DOI:** 10.14202/vetworld.2023.357-368

**Published:** 2023-02-24

**Authors:** Fathia Benlarbi, Nora Mimoune, Noureddine Chaachouay, Karim Souttou, Radhwane Saidi, Mohamed Rahmani Mokhtar, Rachid Kaidi, Mohammed Hocine Benaissa

**Affiliations:** 1 Laboratory for Exploration and Valorization of Steppe Ecosystems (EVES), Department of Biology, Faculty of Natural Sciences and Life, University of Djelfa, Moudjbara Road, BP 3117, Djelfa, Algeria; 2 Department of Clinics, Animal Health and Production Laboratory, Higher National Veterinary School, Algiers, 16000, Algeria; 3 Agri-Food and Health Laboratory, Faculty of Sciences and Techniques of Settat, Hassan FIRST University, Po. Box. 382, 26000 Settat, Morocco; 4 Laboratory of Biological and Agronomic Sciences, Department of Agronomy, Faculty of Sciences, Laghouat University, Laghouat, Algeria; 5 Institute of Veterinary Sciences, LBRA, University of Blida 1, PB 270, Soumaa, Blida, 09000, Algeria; 6 Scientific and Technical Research Centre for Arid Areas (CRSTRA), Biophysical Station, PB 30240, Nezla, Touggourt, Algeria

**Keywords:** antiparasitic medicinal plants, ethnobotanical survey, human and animal parasitosis, Laghouat

## Abstract

**Background and Aim:**

An ethnobotanical survey was carried out among the inhabitants of the Aflou region of Laghouat (Southern Algeria). This study was considered as a first step toward the identification of new bioactive antiparasitic molecules. The preservation and documentation of this traditional knowledge will ensure its continuity and transmission from one generation to another, especially because of the emergence of resistant parasites and the lack of references caused by the lack of work in this area; therefore, we intended to inventory and collect the maximum amount of information on medicinal plants that are traditionally used by the local population as antiparasitic in humans and animals (small ruminants, cattle, and livestock).

**Materials and Methods:**

The information was collected using open interviews; the ethnobotanical survey was carried out in the area mentioned above from April to July 2021 using a semi-structured questionnaire and a global sample of 200 respondents. The data were analyzed using the System Package for the Social Sciences software and Microsoft Excel 2010 using the following quantitative indices: Relative frequency of citation (RFC), family importance value (FIV), fidelity level, and informant consensus factor (ICF).

**Results:**

The investigation uncovered the antiparasitic use of 58 plant species belonging to 30 families. The family *Asteraceae* had the highest FIV (FIV = 0.23). The pathology with the highest degree of agreement among the informants was genitourinary parasitosis (ICF = 0.930). The species that was most commonly cited by the local population was *Artemisia herba-alba* Asso (RFC = 1), and the foliage was the most commonly used part (46.4%). Infusion (38.8%) was the most-used preparation for remedies.

**Conclusion:**

This investigation revealed a rich ethnopharmacological knowledge in southern Algeria; therefore, the data gathered in this survey may be utilized to create novel antiparasitic compounds with activity in humans and animals.

## Introduction

Parasites affecting humans and animals are a severe health complication in developing countries, especially in Africa [1, 2]. More than 1 to 2 billion infections are probably caused by parasites; this causes several million human deaths per year [3]. The appearance of parasitic diseases in humans, such as Chagas disease, malaria, leishmaniasis, schistosomiasis, trypanosomiasis, lymphatic filariasis, helminthic diseases [4], and gastrointestinal parasitosis, is predominantly caused by parasites such as *Haemonchus contortus* and *Fasciola hepatica* from small ruminants [2]. In addition, several ectoparasites, especially ticks, lice, and mites, have also been reported in cattle [5] and humans. Furthermore, parasites cause a decrease in the productivity of the herds, as they reduce fertility, provoke skin irritation, and suck blood, eventually leading to death [6].

In recent years, parasites have been exhibiting resistance to known conventional treatments, which are costly and out of reach for many impoverished individuals [5]. Therefore, it is necessary to discover novel antiparasitic medicinal compounds. Natural products are a crucial source of novel active compounds, because most clinically proven pharmaceuticals are derived from plants [7]. Moreover, traditional knowledge is in danger of extinction. Therefore, the preservation and documentation of this traditional knowledge to revalue this indigenous information are mandatory conditions for maintaining the continuity and transmission of traditional medicine. According to the World Health Organization, traditional medicine is used by 80% of the world’s population to meet their primary healthcare needs [8].

In Algeria, the use of medicinal plants is a thousand-year-old tradition, with more than 4000 species and subspecies of plants being used [9]. Unfortunately, very few ethnobotanical studies have focused on the use of medicinal plants against the predominant internal and external parasites, with the former including *Taenia*, *Oscaris*, *Echinococcus*, *Fasciola* (humans/animals), and *Oxyure* (humans) and the latter including skin leishmania, scabies (skin parasites), ticks, and lice (humans/animals) [10].

In the wilaya of Laghouat, particularly in the Daïra of Aflou, to the best of our knowledge, such references are infrequent because of a lack of work in this direction. This ethnobotanical analysis was carried out among the inhabitants of the Aflou region of Laghouat (Southern Algeria) with the aim of inventorying and collecting as much information as possible on the antiparasitic medicinal plants that are traditionally used by the local population in humans and animals (small ruminants).

## Materials and Methods

### Ethical approval and informed consent

Approval for this study was granted by the Committee for ethical research of the Faculty of Nature and Life Sciences, Department of Biology, Ziane Achour University, with Ref: 012/FSNV/2021. Before starting data collection, oral informed consent was obtained in each case at the site level and then separately before each interview. In addition, informants were made aware that the study’s goals were strictly scientific research and not for commercial purposes. Participants gave verbal consent to participate in the study; they were free to withdraw their information at any time. Finally, informants accepted the topic’s importance and clearly agreed to have their data published without mentioning their names.

### Study period and location

**Figure-1:**
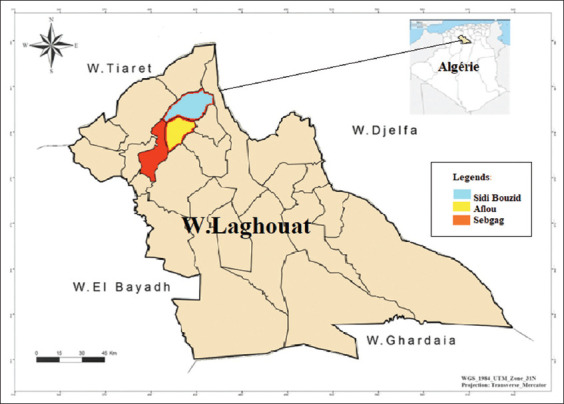
The geographical map of Aflou [Source: https://fr.wikipedia.org/wiki/Da%C3%AFra_d%27Aflou].

The ethnobotanical survey was conducted in Daïra of Aflou from April to July 2021. The Daïra of Afou is an Algerian administrative district located in the Wilaya of Laghouat to the west, 110 km and 406 km from Algiers. The region of Aflou is located in a valley in the heart of the Jebel Amour massif. Built at an altitude of 1400 m, it is one of the highest cities in Algeria. This area is located between (34° 07’ N Latitude and 02° 06’ E Longitude) (Figure-1). The number of inhabitants is 175890 on a total area of 1650 km² [11]. The population density is 106.6 Hab/km² [11]. Aflou has a semi-arid climate; the annual average temperature is 13.42°C with a maximum temperature of 34.37°C in summer) (July) and a minimum of −3.65°C in winter (January), and the rainfall is, on average, 324.38 mm from 2005 to 2014 [12]. The soil is rich in grass and water; it is an area of breeding and grazing, so it is the traditional economic activity of the locality. Aflou, Sebgag, and Sidi Bouzid are the largest municipalities in the Daïra of Aflou, among the three municipalities that compose it. According to the Algerian administrative division and the population density of the commune of Aflou, it is considered an urban area. The other two: Sebgag and Sidi Bouzid are in rural areas; their information is presented in Table-1 [11].

**Table-1:** Distribution of area and density by municipality in the study area [11].

Municipality	Population	Area (km^2^)	Density (habitant/km^2^)
Aflou	160,131	405	395.39
Sidi Bouzid	7,897	860	9.18
Sebgag	7,862	385	20.42

### Data collection

Face-to-face interviews were conducted according to the protocol proposed by Mehdioui and Kahouadji [13]. They were based on discussions performed in the local language using a semi-structured questionnaire that included two parts: One containing general information about the respondent (age, school level, and occupation) and the other concerning the antiparasitic plants used (vernacular name, type of plant, and state of the plant). For the inclusion criteria, we generally targeted local people, herders, traditional healers, and herbalists who had an excellent knowledge of plants for antiparasitic use. In turn, the exclusion criteria were individuals who did not have the necessary knowledge to use medicinal plants and did not live in the study area.

### Sampling and plant species identification

The municipality of Aflou is urban, whereas the remaining two localities (Sidi Bouzid and Sebgag) are rural. The survey allowed us to interview 200 people (27 herbalists, seven traditional practitioners, 100 citizens, and 66 breeders). These respondents were selected by convenience sampling [14]. The determination of the scientific terminology of the local medicinal species collected during the survey was verified and confirmed by the botanists of the Department of Natural and Life Sciences, Faculty of Sciences, University of Laghouat, and with the help of the following bibliographic support [15]. Furthermore, the scientific names of plant species were checked using internet sources; specifically, the list of plants (http://www.theplantlist.org). The ethical guidelines of the International Society of Ethnobiology were adopted [16].

### Data processing

The collected data were entered analyzed by System Package for Social Sciences, version 20 (IBM Corp., NY, USA) and Microsoft Office “Excel 2010”(Microsoft, USA), using descriptive and quantitative statistics of the numbers expressed as percentages. The results of the ethnobotanical survey were analyzed using the relative frequency of citation (RFC), family importance value (FIV), fidelity level (FL), and informant consensus factor (ICF).

### Relative frequency of citation

The RFC value was calculated using the following formula [17]:

RFC = FC/N,

Where, FC is the number of respondents who mentioned the use of the species and N is the total number of respondents. The RFC value varied from 0 (when no individual referred to a plant as applicable) to 1 (when all informants mentioned it as an appropriate medicine).

### Family importance value

The FIV gives local importance to the families of wild species. It is a culturally important metric that can be used in ethnobotany to define the taxonomic value of a natural plant. To calculate the FIV, we used the following formula:

FIV = FC_F_/NS,

where FC_F_ is the number of informants who cited the family and NS is the total number of species in each family [18].

### Fidelity level

The FL factor was calculated using the following formula:

FL (%) = Np/N × 100,

Where, Np is the number of informants who reported the use of the plant species to treat a particular condition and N is the number of informants who used these plants as medicine to treat a given disease [19].

### Informant consensus factor

For data analysis, the ICF was employed to indicate the extent to which the information was homogeneous. Informant consensus factor values will be low (near 0) if plants are chosen randomly, or informants do not exchange information about their use; whereas ICF values will be high (near 1) if there is a well-defined selection criterion in the community and if the information is exchanged among informants. The following formula was used:

ICF = Nur – Nt/Nur – 1 [20],

Where, Nur is the number of citations for each particular condition and Nt is the number of species used to treat that condition.

## Results and Discussion

### Socio-demographic profile of the respondents

A total of 200 local informants, including citizen residents, herbalists, traditional practitioners, and other healers, were interrogated using semi-structured surveys and group interviews (Table-1 and Figure-1).

### Location of the citizens surveyed

The bulk of the local people interviewed (65%) lived in urban regions (Table-2), and most of them were ordinary citizens; the remainder of the interviewees (35%) were breeders residing in rural areas. These proportions are close to those reported by Zougagh *et al*. [14].

**Table-2:** Distribution of 200 respondents by habitat municipality.

Municipalities	Respondents	Percentage
Aflou (urban)	130	65.0
Sebgag (rural)	39	19.5
Sidi Bouzid (rural)	31	15.5

### Gender of the citizens surveyed

The results of our investigation indicated that both sexes are involved in herbal medicine. More than half of the informants were men (58%), including mainly herbalists, breeders, and a large proportion of citizens (Table-3). In comparison, women (42%) were traditional practitioners and the remaining portion of citizens, with a male/female sex ratio of 1.38. This suggests that the profession of herbalist and breeder is preferentially reserved for men. Women’s vigilance can explain this predominance of males for the balance of the disease and their attachment to all that is traditional; males give sustenance and healthcare to their families in the case of an illness. In turn, women practice herbal medicine (traditional practitioners) in the household, which may be attributed to the customs of the region. These results confirm the findings of another ethnobotanical study performed in the Djelfa region [21].

**Table-3:** List of medicinal plants for human and animal parasites in the region of Aflou (Laghouat).

Scientific name and family	Local name	Part used	Preparation form	Administration mode	Traditional uses	FL%	FC	RFC	FIV
Anacardiaceae									0.03
*Pistacia atlantica* Desf.	Butom	Leaves/Galls/Fruit	Infusion/Oil/Decoction	Oral/Swabbing	RA, DA, (H) SP (leish) (H)	50	6	0.03	
Apiaceae									0.029
*Ferula foetida* (Bunge) Regel *Scorodosma poetidum* L.	Hentit	Latex	Maceration	Oral	PD (H)	100	12	0.06	
*Cuminum cyminum* L.	Kamoun	Seeds	Decoction/powder	Oral	PD (H)	100	8	0.04	
*Ferula vesceritensis* Coss.	Fasoukh	Latex	Other	Swabbing	SP (leish) (A)	100	6	0.03	
*Carum carvi* L.	El-Karwia	Seeds	Decoction/powder	Oral	PD (H)	100	2	0.01	
*Bunium mauritanicum* L.	Tal-ghouda	Tuber	Powder	Oral	PD, GD (H)	50	6	0.03	
*Foeniculum vulgare* Mill.	Habet lehlawa	Seeds	Decoction	Oral	PD (H)	100	1	0.005	
Aristolochiaceae									0.035
*Aristolochia baetica* L.	Berrostom	Roots	Powder	Swabbing	SP (leish) (A)	100	7	0.035	
Apocynaceae									0.055
*Nerium oleander* L.	Defla	Leaves/Whole	Infusion/Powder	Swabbing/Rinsing	DA, SP (leish, sca, tic) (A/H)	63.6	11	0.055	
Asteraceae									0.23
*Artemisia herba-alba* Asso	Chih	Leaves/Whole	Infusion/Decoction/Powder	Oral/Rinsing/Swabbing	PD, GP, DA, BP, SP (leish, sca) (A/H)	39.5	200	1	
*Artemisia campestris* L.	Dgouft	Leaves	Infusion	Oral	PD (H)	100	28	0.14	
*Anthemis nobilis* L.	Babounje	Flowers/	Infusion	Rinsing/	DA (H)	100	10	0.05	
*Chamaemelum nobile* L.		Whole		Oral					
*Cotula cinereum* Delile	Guertoufa	Whole	Infusion/Decoction	Oral/Rinsing	PD, DA (H)	93.7	32	0.16	
*Echinacea purpurea* (L.) Moench.	Redbakia	Leaves	Infusion	Oral	GP (H)	100	3	0.015	
*Artemisia absinthium* L.	Chiba	Leaves	Infusion	Oral	PD (A/H)	100	10	0.05	
Brassiaceae									0.035
*Zilla macroptera* Coss.	Chabrag	Whole	Decoction	Rinsing/	DA, SP (leish) (H)	71.4	7	0.035	
Amaranthaceae									0.017
*Hammada scoparia* (Pomel) Iljin	Remth	Whole	Decoction	Rinsing	SP (leish, sca) (A/H)	80	5	0.025	
*Atriplex halimus* L.	Gtaf	Whole	Infusion	Oral	GP (H)		2	0.01	
Cucurbitaceae									0.02
*Cucurbita pepo* L.	Kabouya	Seeds	Raw	Oral	PD (H)	100	2	0.01	
*Colocynthis vulgaris* (L.) schrad.	Hadja	Fruit	Powder	Swabbing	SP (leish, sca) (A/H)	100	7	0.035	
Cupressaceae									0.11
*Juniperus phoenicea* L.	Arar	Whole	Oil/vegetable tar	Swabbing/	DA, AL (A/H) SP (leish, sca, tic) (A/H)	60	25	0.125	
*Cupressus sempervirens* L.	Essarw	Whole	Oil/vegetable tar	Oral/Swabbing/	DA, RA, AL, SP (leish, sca) (A/H)	57.9	19	0.095	
Euphorbiaceae									0.017
*Euphorbia guyoniana* Boiss. and Reut.	Lebina	Stems	Latex	Swabbing/	SP (leish, sca) (H)	100	2	0.01	
*Ricinus communis* L.	Kharoua	Seeds	Oil	Swabbing	AL (H)	100	1	0.005	
Fabaceae									0.025
*Retama raetam* Webb.	Retam	Whole	Decoction/Powder	Oral/Swabbing	SP (leish,sca) (A/H)	100	5	0.025	
Plantaginaceae									0.005
*Globularia alypum* L.	Tasselgha	Leaves	Infusion	Rinsing	SP (sca) (A/H)	100	1	0.005	
Juglandaceae									0.005
*Juglans regia* L.	El-djouz	Leaves	Infusion	Oral	PD (H)	100	1	0.005	
Lamiaceae									0.08
*Thymus guyonii* Noë	Zaatar	Leaves/Whole	Infusion/Decoction	Oral/Rinsing	PD, RA, AL (H) SP (leish) (H)	48.3	29	0.145	
*Thymus ciliatus* Lam.	Djertil	Leaves/Whole	Infusion/Decoction	Oral	PD (A/H)	100	12	0.06	
*Teucrium polium* L.	Djaida	Leaves	Infusion	Oral	PD, BP (H)	58.3	12	0.06	
*Rosmarinus officinalis* L.	Lazir	Leaves	Infusion/Decoction	Oral Swabbing	PD, GP, RA (A/H) AL (H)	45.4	33	0.165	
*Lavandula officinalis* L.	Khzama	Flowers/Leaves	Oil/Infusion	Swabbing/Rinsing	GP, AL (A/H)	90.9	33	0.165	
*Salvia officinalis* L.	Miramia	Leaves	Infusion	Oral	GD (H)	100	8	0.04	
*Origanum majorana* L.	Bardakouch	Leaves	Infusion	Oral	GD (H)	100	12	0.06	
*Mentha spicata* L.	Naanaa	Leaves	Infusion/Oil	Oral	RA, PD (H)	66.7	3	0.015	
*Mentha pulegium* L.	Feliou	Leaves	Infusion	Oral	PD (H)	100	2	0.01	
Lauraceae									0.01
*Cinnamomum verum* J.Presl	Qarfa	Bark of trunk	Decoction	Oral	RA (H)	100	2	0.01	
Amaryllidaceae									0.188
*Allium sativium* L.	Toum	Clove	Raw	Oral/Swabbing	PD, AL, SP (leish, sca) (A/H)	42.6	61	0.305	
*Allium cepa* L.	Basla	Clove	Raw/Maceration	Oral/Swabbing	PD, AL (A/H)	71.4	14	0.07	
Xanthorrhoeaceae									
*Aloe vera* (L.) Burm.f.	Mor -sebar	GÈLE/suc	Powder	Oral/Swabbing	PD, SP (leish, sca) (A/H)	66.7	3	0.015	0.015
Linaceae									0.005
*Linum usitatissimum* L.	Zereat-ketane	Seeds	Decoction	Oral	AL (H)	100	1	0.005	
Lythraceae									0.02
*Lawsonia inermis* L.	Elhénna	Leaves	Powder	Swabbing	SP (leish, sca) (A/H)	100	4	0.02	
Meliaceae									0.05
*Melia azedarach* L.	Mélia/Sébahiya	Leaves/Fruit	Decoction/Oil/Lotion	Oral/Swabbing/Rinsing	SP (leish, sca) PD, AL (A/H) Insecticid	60	10	0.05	
Myrtaceae									0.038
*Eugenia caryophyllus* L.	Lekrounfel	Flower bud	Decoction/Oil	Oral/Swabbing	SP (leish, sca) (H) PD, AL (H)	50	6	0.03	
*Eucalyptus globulus* Labill.	Kalitousse	Leaves	Infusion/Decoction	Oral/Rinsing	DA, RA (H) AL, BP (H)	46.7	15	0.075	
*Myrtus communis* L.	Rihane	Leaves	Infusion	Oral	PD (H)	100	2	0.01	
Oleaceae									0.05
*Olea europea* L.	Zitoun	Fruit/Leaves	Oil/Infusion	Oral/Swabbing	DA (A/H)	100	10	0.05	
Piperaceae									0.005
*Piper nigrum* L.	Felfel-akhal	Seeds	Powder	Swabbing	AL (H)	100	1	0.005	
Punicaceae									0.075
*Punica granatum* L.	Romane	Bark of the fruit	Raw/Decoction	Oral	PD (A/H)	100	15	0.075	
Renonculaceae									0.015
*Nigella sativa* L.	Sanoudj	Seeds	Decoction/Oil	Oral/Swabbing	PD (A/H) BP (H)	66.7	3	0.015	
*Hydrastis canadensis* L.	Khatem-dehab	Rhizom	Decoction	Oral	GP (H)	100	3	0.015	
Rhamnaceae									0.05
*Zizyphus lotus* (L.) Lam.	Sedra	Leaves	Powder	Swabbing	SP (leish, sca) (H) DA (A/H)	80	10	0.05	
Rutaceae									0.045
*Citrus limon* (L.) Osbeck	Laymoune	Fruit	Maceration	Oral	PD (A/H)	100	14	0.07	
*Ruta graveolens* L.	Fidjel	Whole	Infusion/Powder	Oral/Swabbing	PD (H) AL (H)	50	4	0.02	
Theaceae									0.025
*Camellia sinensis* (L.) Kuntze	Latay	Leaves	Decoction	Oral	PD (A)	100	5	0.025	
Zingiberaceae									0.072
*Zingiber officiale* Roscoe	Zandjabil	Rhizom	Raw/Powder	Oral	PD, RA (A/H) GP (H)	41.6	24	0.12	
*Curcuma longa* L.	Kourkoum	Rhizom	Powder/Maceration	Oral	PD (H)	100	5	0.025	
Zygophyliaceae									0.055
*Peganum harmala* L.	Harmal	Seeds	Powder/Decoction	Oral Swabbing	SP (leish, sca) (H) AL (H) BP (H)	54.5	11	0.055	

PD=Parasites of the digestive tract, SP (sca)=Skin Parasites (scabies), SP (leish)=Skin Parasites (leichmaniasis), SP (tic)=Skin Parasites (ticks), DA=Dermatologic Affection; GP=Genetourinary Parasites, RA=Respiratory ailments, GD=Gland Disorders, BP=Blood Parasites (malaria), A/H=Animals/humans, AL=Affection by Lice, FL=Fidelity level, FC=Frequency citation, RFC=Relative frequency of citation, FIV=Family importance value

### Age of the citizens surveyed

**Figure-2:**
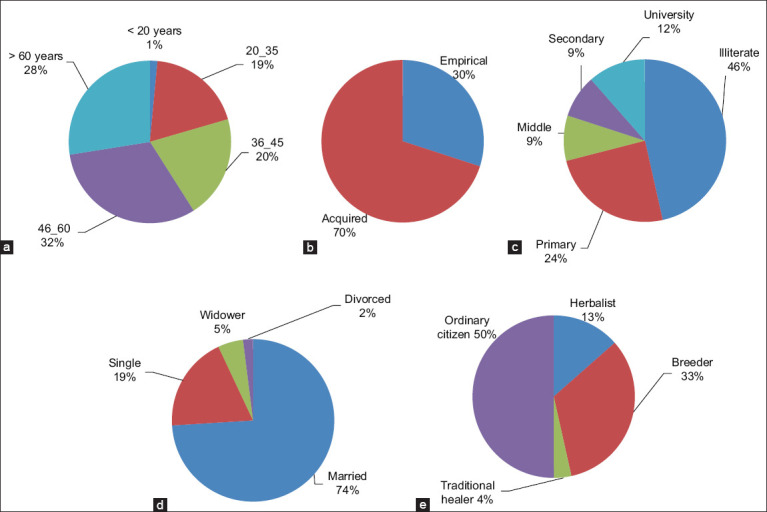
Socio-demographic profile of the informants; (a) Age of the citizens surveyed (b) Origin of information of the respondents (c) Educational level of the respondents (d) Marital status of the respondents and (e) Occupation of the respondents.

The findings of this investigation revealed that the use of medicinal species is prevalent across all age classes, with varying percentages. The majority of the respondents were in the age range between 40 and 60 years (31.3%), followed by informants who were older than 60 years (27.5%), informants who were between 36 and 45 years (20.5%), and informants who were between 20 and 35 years (19%). Finally, informants younger than 20 years came in the last position (1.5%) (Figure-2a). The data showed that the elderly acquired therapeutic knowledge from their parents or the experiences of others. The oldest informants offer more credible information because they possess much of the traditional knowledge that is part of the folk tales. As a result, there is a loss of knowledge about medicinal plants, which may be explained by the doubts of some young people who are disinterested in herbal medicine because of modernization and foreign cultural influences. Furthermore, knowledge of the properties and use of medicinal plants is often acquired (70%) through a long-accumulated experience (Figure-2b), and then passed orally from one generation to another [13, 22].

### Educational level of the respondents

Regarding educational level, 46.5% of the respondents were illiterate, 24.5% had a primary school level, 17.5% were between a middle and secondary school level, and 11.5% had a university level (Figure-2c). Thus, the use of medicinal plants decreased as the level of education increased [23]. Our results confirmed the findings of the study performed by Bouzid *et al*. [23], who reported that more than half of the users of medicinal plants (64%) were illiterate and 27% of the users had a primary school level. In contrast, very few of those with a university level (9%) used medicinal plants [22].

### Marital situation of the respondents

In terms of marital status, married respondents (74%) used medicinal plants much more than did single respondents (19%), with 05 % of widowers versus only 2% of divorced informants using these plants (Figure-2d). The difference between family status and Indigenous knowledge for treating antiparasitic infections was statistically significant (p = 0.000). This may be explained by the fact that married individuals may avoid or reduce the material fees used to pay doctors and pharmacists [21, 22].

### Occupation of the respondents

Half of the respondents interviewed in the study area were ordinary citizens who used antiparasitic plants to remedy their illnesses. Concomitantly, breeders accounted for only 33% of the respondents, with the remainder of the cohort being distributed between herbalists and traditional practitioners (Figure-2e).

### Plants surveyed

#### Most-used botanical families

**Figure-3:**
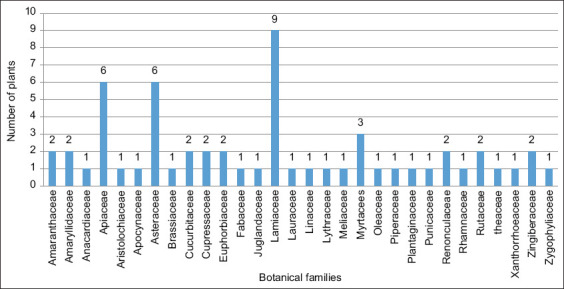
The most-used botanical families.

The data collected allowed the identification of 58 species of plants belonging to 30 botanical families that were used to treat parasitic diseases in humans and animals (small ruminants). These plants are presented in Table-4 using their family and scientific names, local names, parts used, the form of preparation, mode of administration, and quantitative values (FL, ICF, RFC, and FIV). The most represented families were *Lamiaceae*, with nine species; followed by *Asteraceae* and *Apiaceae*, six species each; and *Myrtaceae*, three species. In comparison, the remaining families were represented by two or only one species (Figure-3). According to research performed in the Mediterranean region, *Lamiaceae* and *Asteraceae* are the most-used plants in traditional medicine [10, 14, 24]. *Asteraceae* (FIV = 0.23), *Amaryllidaceae* (FIV = 0.188), *Cupressaceae* (FIV = 0.11), and *Lamiaceae* (FIV = 0.08) (Table-3) were the four families most cited according to the FIV index. This high proportion could be explained by the fact that these families are widely present among the flora of Aflou. This dominance was also observed, albeit with some differences, in the results of other ethnobotanical surveys conducted in other countries [14, 22].

**Table-4:** Informant consensus factor values by category for treating parasitosis.

Pathology	Plants species and number of uses	Nt	Nur	ICF
Genitourinary parasites	*Artemisia herba-alba* Asso. (29), *Echinacea purpurea* (L.) Moench (3), *Hydrastis canadensis* L. (3), *Cupressus sempervirens* L. (11), *Rosmarinus officinalis* L. (15), *Lavandula officinalis* L. (30), *Zingiber officiale* Roscoe. (8), *Atriplex halimus* L. (2).	8	101	0.930
Blood parasites	*Artemisia herba-alba* Asso. (45), *Teucrium polium* L. (5), *Eucalyptus globulus* Labill. (4), *Nigella sativa* L. (2), *Peganum harmala* L. (1).	5	57	0.929
Gland disorders	*Bunium mauritanicum* L. (3), *Origanum majorana* L. (12), *Salvia officinalis* L. (8).	3	23	0.909
Dermatological affection	*Pistacia atlantica* (Desf). (3), *Nerium oleander* L. (7), *Artemisia herba-alba* Asso. (12), *Anthemis nobilis* L. (10), *Cotula cinereum* Delile (30), *Eucalyptus globulus* Labill. (7), *Zilla macroptera* Coss. (5), *Olea europea* L. (10), *Zizyphus lotus* (L.) Lam. (8), *Peganum harmala* L. (6), *Cupressus sempervirens* L. (3), *Juniperus phoenicea* L. (6).	12	107	0.896
Parasites of the digestive tract	*Ferula foetida* (Bunge) Regel (12), *Cuminum cyminum* L. (8), *Carum carvi* L. (2), *Bunium mauritanicum* L. (3), *Artemisia herba-alba* Asso. (79), *Foeniculum vulgare* Mill. (1), *Artemisia campestris* L. (28), *Cotula cinereum* Delile (2), *Artemisia absinthium* L. (10), *Cucurbita pepo* L. (2), *Juglans regia* L. (1), *Allium sativium* L. (10), *Allium cepa* L. (4), *Camellia sinensis* (L.) Kuntze (5), *Aloe vera* (L.) Burm.f. (1), *Thymus guyonii* Noë (14), *Thymus ciliatus* (Desf). (12), *Teucrium polium* L. (7), *Mentha spicata* L. (2), *Mentha pulegium* L. (2), *Rosmarinus officinalis* L. (5), *Melia* *azedarach* L. (1), *Eugenia caryophyllus* L. (1), *Myrtus communis* L. (2), *Punica granatum* L. (15), *Nigella sativa* L. (1), *Citrus limon* (L.) Osbeck (14), *Zingiber officiale* Roscoe. (6), *Curcuma* *longa* L. (5), *Ruta graveolens* L. (2).	30	257	0.887
Affections by Lice	*Juniperus phoenicea* L. (15), *Cupressus sempervirens* L. (3), *Ricinus communis* L. (1), *Allium* *sativium* L. (25), *Allium cepa* L. (10), *Thymus guyonii* Noë (8), *Lavandula officinalis* L. (3), *Linum usitatissimum* L. (1), *Melia azedarach* L. (6), *Eugenia caryophyllus* L. (3), *Eucalyptus* *globulus* Labill. (2), *Piper nigrum* L. (1), *Ruta graveolens* L. (2), *Rosmarinus officinalis* L. (10), *Peganum harmala* L. (2).	15	92	0.846
Skin parasite	*Pistacia atlantica* (Desf). (2), *Ferula vesceritensis* Coss. (6), *Artemisia herba-alba* Asso. (35), *Hammada scoparia* (Pomel) Iljin. (5), *Colocynthis vulgaris* (L.) schrad. (7), *Juniperus phoenicea* L. (4), *Euphorbia guyoniana* Boiss. & Reut. (2), *Zilla macroptera* Coss. (2), Retama *raetam* Webb. (5), *Globularia alypum* L. (1), *Allium sativium* L. (26), *Aloe vera* (L.) Burm.f. (2), *Thymus guyonii* Noë (5), *Melia azedarach* L. (3), *Eugenia caryophyllus* L. (1), *Zizyphus lotus* (L.) Lam. (2), *Lawsonia inermis* L. (4), *Peganum harmala* L. (2), *Cupressus sempervirens* L. (2), *Nerium oleander* L. (4), *Aristolochia baetica* L. (7).	21	127	0.841
Respiratory ailments	*Pistacia atlantica* (Desf). (1), *Thymus guyonii* Noë (2), *Rosmarinus officinalis* L. (3), *Cinnamomum verum* J.Presl. (2), *Eucalyptus globulus* Labill. (2), *Zingiber officiale* Roscoe. (10), *Mentha* *spicata* L. (1), *Eugenia caryophyllus* L. (1).	8	22	0.666

ICF=Informant consensus factors, Nur=Number of citations for each particular condition, Nt=Number of species used to treat that condition.

#### Most frequently cited medicinal plants

Some of the antiparasitic medicinal plants that were inventoried during the investigations were more frequently cited in the Aflou region. The RFC of the medicinal plants cited ranged from 0.005 to 1 (Table-3). The highest values were observed for *Artemisia herba-alba* Asso (RFC = 1), followed by *Allium sativium* L. (RFC = 0.305), *Rosmarinus officinalis* L. (RFC = 0.165), *Lavandula officinalis* L. (RFC = 0.165), *Cotula cinereum* Delile (RFC = 0.16), *Thymus guyonii Noë* (RFC = 0.145), *Artemisia campestris* L. (RFC = 0.14), *Juniperus phoenicea* L. (RFC = 0.125), *Zingiber officinale* Roscoe (RFC = 0.12), *Punica granatum* L. (RFC = 0.075), *Allium cepa* L. (RFC = 0.07), *Artemisia absinthium* L. (RFC = 0.05), and *Olea europea* L. (RFC = 0.05). Their high RFC values indicate that these plants are the most known and used by most respondents to treat parasitic diseases.

Many investigations aimed at testing their biological and phytochemical activity have been carried out for these plants. In India, Singh *et al*. [25] reported that *Allium sativum* L. is used against amoebiasis and as a dewormer in animals [26]. *Artemisia herba-alba*, *T. guyonii* Noë, *J. phoenicea* L., and *A. campestris* L. are used as antiparasitic plant remedies in Algeria, as reported by Boudjelal *et al*. [10]. Kpabi *et al*. [27] found that *Zingiber* *officinale* Roscoe is used against amoebiasis in northern Togo. Several studies have mentioned the use of *L. officinalis* L. to control lice and other external parasites [28]. In Morocco, El Rhaffari and Zaid [29] proved the anti-leishmania activity of *R. officinalis* L., and veterinarians continue using it as a pulmonary antiseptic in animals [26]. Regarding *J. phoenicea* L., the most-used form of preparation of this plant is juniper oil, which is obtained after the distillation of the wood of old trees. This oil is also known as cade oil, vegetable tar, or by the Arabic name of Gatran. It is mainly a veterinary product that is used to treat specific animal diseases in the study area: fasciolosis and as a deworm for sheep [26]. It was associated with *Olea europea* L. for use in humans and particularly to remove external parasites on domestic animals [30]. Root macerations of *P. granatum* L. are used to control tapeworms in small ruminants [26] and against roundworms [31]. *Allium* *cepa* L. and *Artemisia absinthium* L. are also used as anthelmintics in humans and animals [26]. The medicinal properties of *Artemisia herba-alba* Asso and *A. campestris* L. remain of interest to researchers [32].

### Fidelity level index

According to our results, most plants had a high FL, with a value of 100% recorded for 25 plant species (Table-3). Most of them were used by several informants to treat a single pathology, and we ignored the plants that were mentioned only once for better accuracy [22]. The high FL of a species indicates the presence of a specific disease in a given area and the use of plant species to treat it by its citizens [33]. Therefore, the plant species with the highest FL that had not been previously researched should be suggested for future clinical-practice-related investigations.

### Pathologies and their ICF values

The ICF ranges from 0 to 1. A high ICF indicates agreement regarding plant species selection among informants, whereas a low value indicates disagreement. Recently, a consensus ratio analysis was used as an important factor for ethnobotanical data analysis [22, 34]. The ICF values in this study ranged from 0.666 to 0.930, depending on the pathology treated (Table-4). The condition with the highest level of agreement among informants was genital–urinary parasites (0.930), followed by blood parasites (0.929), glandular conditions (0.909), dermatological conditions (0.896), digestive tract parasites (0.887), lice-associated conditions (0.846), skin parasites (0.841), and respiratory diseases (0.666). These high ICF values indicate reasonable reliability in the use of medicinal plant species by citizens [35]. Moreover, they demonstrate the most significant agreement between medicinal plants and parasitic diseases because the informants often utilized certain plant species to treat antiparasitic disorders.

### Parts of plants used, method of preparation, and routes of administration of recipes

#### Parts of plants used

**Figure-4:**
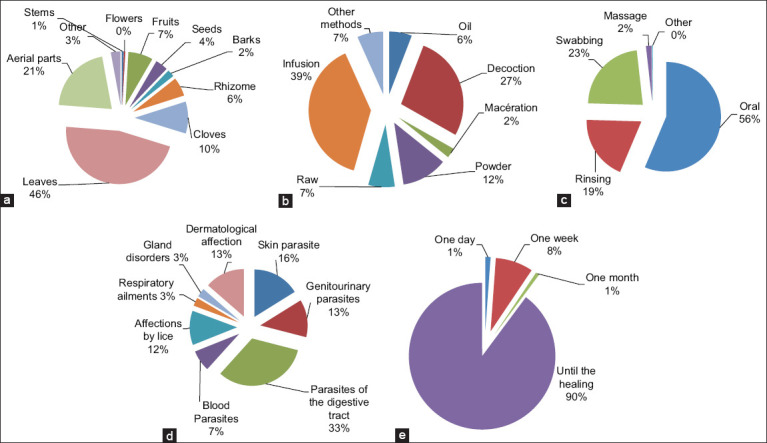
Antiparasitic medicinal plants and their different criteria; (a) Parts of plants used (b) Forms of treatment preparations (c) Modes of administration (d) Diseases treated and (e) Duration of treatment.

Our results showed that the foliage is the most-used plant part, with a percentage of 46.4%, followed by whole plants (20.9%), bulbs (9.7%), fruits (7.5%), rhizomes (5.7%), and seeds (3.8%). The least used parts are barks (2.2%), stems (0.5%), and flowers (0.4%) (Figure-4a). We also found that other parts of plants, such as gels, juices, or latex, were used at a percentage of 2.9% in total. Thus, the leaves are the most-used plant organs in the preparation of remedies in the Aflou region. Similar results indicated that leaves were the most dominant plant parts [36, 37]. This could be because the leaves are sites of photochemical reactions [38] and are characterized by ease and speed of harvesting [39]. The fight against overgrazing can be promoted by applying the technique of prohibiting plowing in pastoral areas. The census of endangered plants and the encouragement of specialized state nurseries to produce plants and distribute them to environmental protection associations for planting should be promoted.

#### Form of preparation

The parts of antiparasitic plants in the study area were prepared in the form of infusion (38.8%), followed by decoction (27.4%), powder (11.8%), raw (6.9%), maceration (2.5%), and other forms of preparation (6.7%) (Figure-4b). The predominant use of the infusion form can be explained by the fact that this technique allows the extraction of the greatest number of active principles and attenuates or cancels the toxic effects of specific traditional recipes. Several other ethnobotanical studies have shown that most respondents prepared the remedies by infusion [22, 40].

#### Method of administration

The route of administration is related to the type of pathology to be treated and the form of preparation of the plants used. In general, the traditional recipes prepared were administered through the oral route (56.4%), because it is the most straightforward, most effective, and quickest route, followed by brushing (22.8%), rinsing (19.1%), and massage in only 1.7% of cases. Most ethnobotanical studies conducted in Africa [41–43] agree with our results regarding the predominance of oral administration (Figure-4c).

### Type of disease treated

The ethnobotanical investigation conducted in Aflou listed some parasitic diseases treated by medicinal plants, with the most frequent being (in descending order): Parasites of the digestive tract and its annexes (32.7%), cutaneous parasites (16.2%), dermatological affections (13.6%), genital–urinary parasites (12.8%), affections caused by lice (11.7%), and blood parasites (7.2%). The other diseases treated, such as respiratory and glandular disorders, did not exceed 3% (Figure-4d). Diseases of the digestive tract were the most widely treated by medicinal plants. These results are similar to those reported in Africa [36, 44].

### Duration of treatment

The treatment duration varied according to the pathologies to be treated and the plants used. This duration can be indefinite and will continue until the patient is cured; thus, it can be limited to 1 day or will extend to 1 week or even 1 month. Our results were similar to those of Slimani *et al*. [44] and Yabrir *et al*. [21], who reported that the duration of treatment continued until the illness was cured (89.8%) (Figure-4e).

## Conclusion

The present study was the first of its kind, revealing that 58 plant species were employed for treating parasitosis in humans and animals (small ruminants) among the inhabitants surveyed in the region of Aflou in Southern Algeria. Moreover, 18 of these species were introduced species that were imported from other regions or countries, such as *Ferula foetida* (Bunge) Regel, *Cuminum cyminum* L., *Ferula vesceritensis* Coss, *Carum* *carvi* L., *Foeniculum vulgare* Mill., *Aristolochia baetica* L., *Echinacea purpurea* (L.) Moench, *Salvia officinalis* L, *Origanum majorana* L., *Cinnamomum verum* J. Presl, *Linum usitatissimum* L., *Eugenia caryophyllus* L., *Piper nigrum* L., *Nigella sativa* L., *Hydrastis Canadensis* L., *Camellia sinensis* (L.) Kuntze, *Zingiber officinale* Roscoe, and *Curcuma longa* L.; however, the majority of the species reported in this survey were native plants. The ethnobotanical survey revealed the incredible biodiversity of the medicinal and aromatic antiparasitic plants used in the region. These plants are prevalent and widely used. We also observed that the information collected through this survey regarding traditional medicine was mainly restricted to the elderly. There is a risk that this knowledge will be lost before it is passed on to future generations. Therefore, the preservation and documentation of this traditional knowledge are necessary for maintaining the continuity and transmission of traditional medicine. Our survey was considered as a first step toward the completion of the research and evaluation of the actual efficiency of the mentioned plants. This could lead to the development of new antiparasitic molecules that are active in humans and animals.

## Authors’ Contributions

FB, RS, KS, and NC: Conceptualization, investigation, methodology, and writing - original draft. NM and MRM: Visualization, supervision, and interpretation of the data. RK, RS, and MHB: Supervision, methodology, and writing - review and editing. All authors have read, reviewed, and approved the final manuscript.
